# Androgen receptor non-nuclear regulation of prostate cancer cell invasion mediated by Src and matriptase

**DOI:** 10.18632/oncotarget.3119

**Published:** 2015-01-29

**Authors:** Jelani C. Zarif, Laura E. Lamb, Veronique S. Schulz, Eric A. Nollet, Cindy K. Miranti

**Affiliations:** ^1^ Laboratory of Integrin Signaling and Tumorigenesis, Van Andel Research Institute, Grand Rapids, MI 49503, USA; ^2^ Cell and Molecular Biology Program, Michigan State University, East Lansing, MI 48824, USA; ^3^ Department of Urology, Beaumont Health System - Research Institute, Royal Oak, MI 48073, USA; ^4^ Van Andel Institute Graduate School, Grand Rapids, MI 49503, USA

**Keywords:** prostate cancer, nongenomic AR signaling, Src, metastasis, castration-resistant

## Abstract

Castration-resistant prostate cancers still depend on nuclear androgen receptor (AR) function despite their lack of dependence on exogenous androgen. Second generation anti-androgen therapies are more efficient at blocking nuclear AR; however resistant tumors still develop. Recent studies indicate Src is highly active in these resistant tumors. By manipulating AR activity in several different prostate cancer cell lines through RNAi, drug treatment, and the use of a nuclear-deficient AR mutant, we demonstrate that androgen acting on cytoplasmic AR rapidly stimulates Src tyrosine kinase via a non-genomic mechanism. Cytoplasmic AR, acting through Src enhances laminin integrin-dependent invasion. Active Matriptase, which cleaves laminin, is elevated within minutes after androgen stimulation, and is subsequently shed into the medium. Matriptase activation and shedding induced by cytoplasmic AR is dependent on Src. Concomitantly, CDCP1/gp140, a Matriptase and Src substrate that controls integrin-based migration, is activated. However, only inhibition of Matriptase, but not CDCP1, suppresses the AR/Src-dependent increase in invasion. Matriptase, present in conditioned medium from AR-stimulated cells, is sufficient to enhance invasion in the absence of androgen. Thus, invasion is stimulated by a rapid but sustained increase in Src activity, mediated non-genomically by cytoplasmic AR, leading to rapid activation and shedding of the laminin protease Matriptase.

## INTRODUCTION

The survival of malignant tumors arising from the prostate gland is dependent on the androgen receptor (AR), a classical nuclear steroid receptor that binds androgen and activates gene transcription [1, 2]. This dependence on androgen is exploited therapeutically; patients presenting with metastatic disease are treated with anti-androgen therapies that effectively lower circulating androgen levels and cause tumor regression. However, patients typically relapse within one to two years and develop castration-resistant disease in which the tumors no longer respond to androgen ablation therapy [3]. Nonetheless, AR itself is still critical as demonstrated by its retention, mutation, and amplification in resistant disease [4, 5]. The critical role of AR is further supported by the fact that more potent second generation anti-androgen therapies are able to extend patient survival. Unfortunately, even these therapies are still not curative.

Studies with the new anti-androgen agent MDV3100 (enzalutamide) indicate one of the ways it inactivates AR nuclear activity in the tumor cells is by preventing nuclear translocation, thus retaining a significant amount of AR the cytoplasm [6, 7]. Several steroid receptors, including AR, are reported to have non-nuclear (aka non-genomic) signaling functions in the cytoplasm independent of their transcriptional activity [8–13]. These non-nuclear signaling mechanisms are associated with rapid responses (within minutes) of steroid stimulation. One common target of steroid receptor nongenomic signaling is the non-receptor tyrosine kinase, Src [14–17]. Activated Src correlates with resistance to MDV3100 (enzalutamide) in castration-resistant patients [18]. Thus, it is necessary to understand how Src promotes castration-resistant disease.

Src is a prototypic member of the non-receptor protein tyrosine kinase family (SFK) up-regulated or hyper-activated in a high percentage of human cancers [19, 20]. SFK activation and signaling is highly associated with bone metastasis in breast and prostate cancer [21, 22]. Inhibition of Src and Lyn with dasatinib decreased prostate cancer growth and lymph node metastasis in AR-independent and -dependent xenograft models [23]. *In vitro*, Lyn was required primarily for tumor cell growth, while Src was responsible for promoting cell migration. The specific effect of Src on migration is not surprising given that many Src substrates, such as FAK, Cas, and cortactin, are intimately involved in promoting cell motility through integrin-mediated adhesion to extracellular matrices [17, 21].

Src is activated nongenomically in cells by progesterone receptor (PR), estrogen receptor (ER), and AR, via interactions between the Src SH3 domain and a PXXP-containing scaffold protein, NMAR/PELP, that binds steroid receptors via LXXLL motifs. Alternatively, the Src SH3 domain binds directly to the PXXP motif in AR or PR [14, 15]. Assembly of Src-containing ER and AR complexes activates MAPK signaling to stimulate cell proliferation and castration-resistance [24, 25]. ER, acting through Src to activate ILK1 was recently shown to be responsible for enhancing breast cancer invasion and metastasis [26]. While several studies independently linked increased invasion and metastasis with elevated AR or Src activity [27, 28], the specific role of cytoplasmic AR signaling via Src to promote prostate cancer migration and invasion was not reported.

We previously demonstrated that AR activation in prostate cancer cells increases integrin α6β1 transcription and expression [29, 30]. During that study, we also observed that AR activation induced morphological changes in the cells. We hypothesized that AR contributes to prostate cancer cell migration and invasion, possibly through stimulation of integrin α6β1 transcription. As described herein, we identified an AR-dependent non-nuclear signaling pathway, independent of integrin α6β1 transcription, leading to Src activation and subsequent cleavage and shedding of Matriptase, required for prostate cancer invasion.

## RESULTS

### AR stimulation alters cell shape, migration, and invasion via laminin integrins

**Figure 1:**
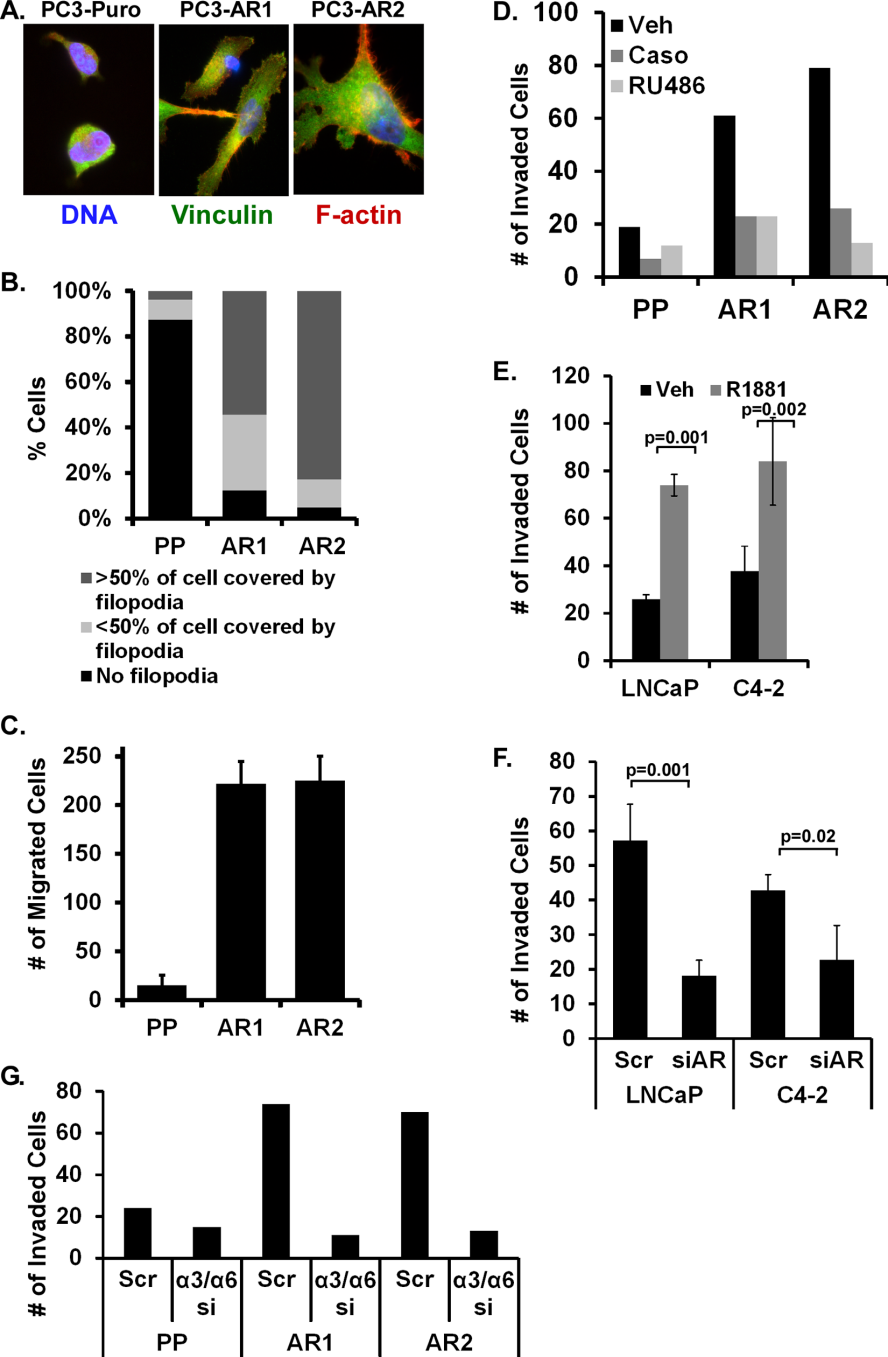
AR stimulation alters cell shape, migration, and invasion via laminin integrins **(A, B)** Parental PC3 (Puro) and 2 PC3-AR clones (AR1 and AR2) were plated on laminin for 1 hour then (A) immunostained for vinculin (green), stained for F-actin with phalloidin (red), and counterstained with Hoecsht (blue). (B) Percentage of cells with filopodia was quantified. **(C)** Migration on laminin-coated Boyden chamber inserts was quantified in PC3-Puro (PP) versus PC3-AR clones. **(D)** Extent of invasion through Matrigel-coated Boyden chambers was quantified following treatment of PC3 (Puro) or PC3-AR clones with ethanol (Veh), 10 nM Casodex (Caso), or 10 nM RU486. **(E, F)** Matrigel invasion was measured in LNCaP or C4–2 cells treated with ethanol (Veh) or 10 nM R1881 for 24 hours (E) without or (F) with scrambled siRNA (Scrm) or AR-specific siRNA (siAR). **(G)** Matrigel invasion by PC3-Puro (PP) or PC3-AR clones treated with scrambled (Scr) or combined integrin α3 and α6 siRNA (a3/a6) was measured.

Previously, we generated PC3 cells stably expressing wild type AR [29]. We demonstrated AR was constitutively nuclear localized and activated in PC3-AR cells as measured by immunofluorescent staining and PSA expression. During those studies we observed marked changes in cell morphology in the AR-expressing cells compared to the parental vector cells (PC3-Puro). To quantify these differences, PC3-Puro and PC3-AR cells were plated on laminin and immunostained to visualize actin structures. There was a marked increase in cell spreading on laminin by the AR-expressing cells compared to the vector cells (Figure 1A), which was accompanied by an 8-fold increase in filopodial structures (Figure 1B). The observed increase in filopodia formation specifically in PC3-AR cells correlated with a 22-fold increase in Boyden chamber migration (Figure 1C) as well as a 3- to 4-fold increase in Matrigel invasion (Figure 1D) compared to PC3-Puro vector cells. Furthermore, inhibiting AR activity with Casodex or RU486 attenuated the AR-specific increase in Matrigel invasion (Figure 1D). Complementary experiments were conducted using LNCaP and C4–2 cells which express endogenous AR. In LNCaP and C4–2 cells, stimulation of AR with R1881 increased Matrigel invasion 3- and 2.4-fold respectively (Figure 1E). Conversely, AR suppression using siRNA decreased Matrigel invasion stimulated by R1881 (Figure 1F). We previously demonstrated that AR transcriptionally induces integrin α6β1 expression [29]. However, blocking integrin α6 or α3 expression alone did not block invasion (not shown). Depletion of both laminin integrins, α3 and α6, was required to suppress Matrigel invasion 4-fold (Figure 1G). Thus AR promotes the migration and invasion of prostate tumor cells through Matrigel via either of two laminin-specific integrins.

### AR stimulates Src activation

**Figure 2:**
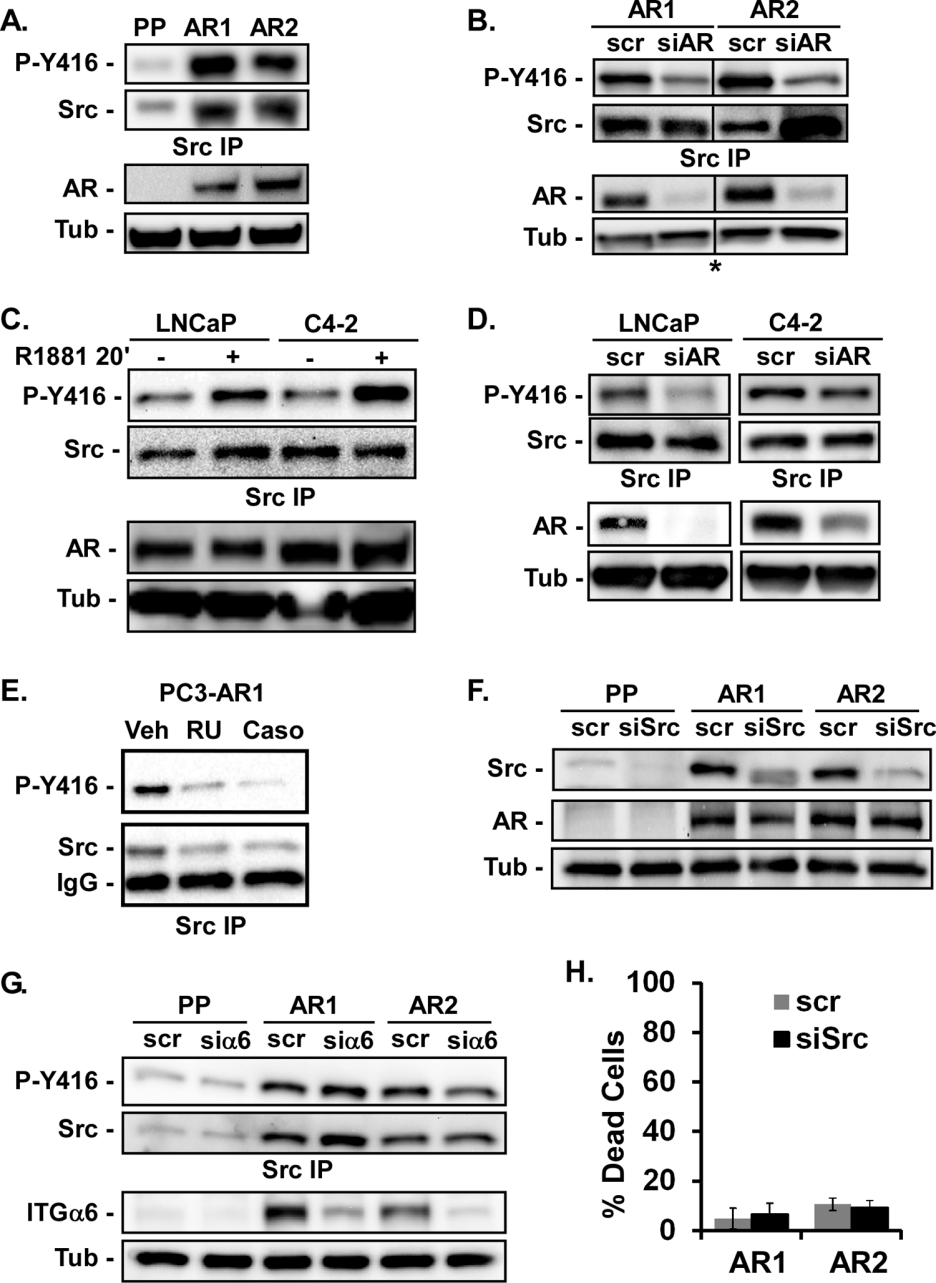
AR stimulates Src activation **(A, B)** Src activation and expression in PC3-Puro (PP) or PC3-AR clones (AR1/AR2) was measured by immunoblotting of Src immunoprecipitates with anti-Y416 phospho-specific antibody or total anti-Src antibody respectively in (A) untreated cells or (B) cells treated with scrambled siRNA (scr) or AR-specific siRNA (siAR). *All samples were run on the same gel, but image was cut to remove extraneous lanes. Total levels of AR and tubulin (Tub) expression served as loading controls and were monitored by immunoblotting. **(C, D)** Src activation and expression was measured in LNCaP or C4–2 cells stimulated with ethanol (−) or 10 nM R1881 for 20 minutes in (C) untreated cells or (D) cells treated with scrambled siRNA (scr) or AR-specific siRNA (siAR). **(E)** Src activation and expression was measured in PC3-AR1 cells treated with ethanol (Veh), 10 nM RU486 or 10 nM Casodex (Caso) for 24 hours. **(F)** Src and AR expression were measured by immunoblotting in PC3-Puro (PP) or PC3-AR clones (AR1/AR2) treated with scrambled siRNA (scr) or Src-specific siRNA (siSrc). **(G)** PC3-Puro (PP) or PC3-AR clones (AR1/AR2) were treated with scrambled siRNA (scr) or integrin α6-specific siRNA (siα6). Src activation and expression in immunoprecipitates and integrin α6 expression cell lysates were measured by immunoblotting. **(H)** Percentage of cell death was measured by trypan blue dye exclusion following adhesion to laminin.

Src is a major effector of cell spreading, migration, and invasion [19, 20]. Therefore we investigated Src activation and expression in the PC3-Puro and PC3-AR cells. We found elevated Src expression as well as increased Src activity in PC3-AR cells as measured by anti-phospho-[Y^416^]-Src antibody (Figure 2A). Inhibiting AR expression in PC3-AR clones with siRNA decreased Src activation, but not total Src (Figure 2B) demonstrating AR regulates Src activation but not its expression. Stimulating LNCaP or C4–2 cells with R1881 for twenty minutes was sufficient to increase Src activation, but did not change Src expression (Figure 2C). Conversely, inhibiting AR expression with siRNA in LNCaP and C4–2 cells suppressed Src activation in response to R1881 (Figure 2D). Similarly, suppressing AR activation in PC3-AR cells with RU486 or Casodex suppressed Src activation (Figure 2E). Thus, AR is required for Src activation in several different prostate tumor cell lines.

Inhibiting Src with siRNA did not alter AR expression (Figure 2F) indicating Src is downstream of AR. AR stimulates integrin α6 [29], which could activate Src. However, inhibiting integrin α6 expression with siRNA did not change Src activity or expression (Figure 2G). Src is also not involved in AR-dependent survival signaling, since suppressing Src expression with siRNA in PC3-AR clones caused no increase in cell death (Figure 2H). Treatment of PC3-Puro or PC3-AR cells with RU486 or Casodex also did not induce cell death (not shown). Thus AR, independently of its actions on integrin α6 expression or cell survival, stimulates Src activity.

### AR and Src are required for invasion

**Figure 3:**
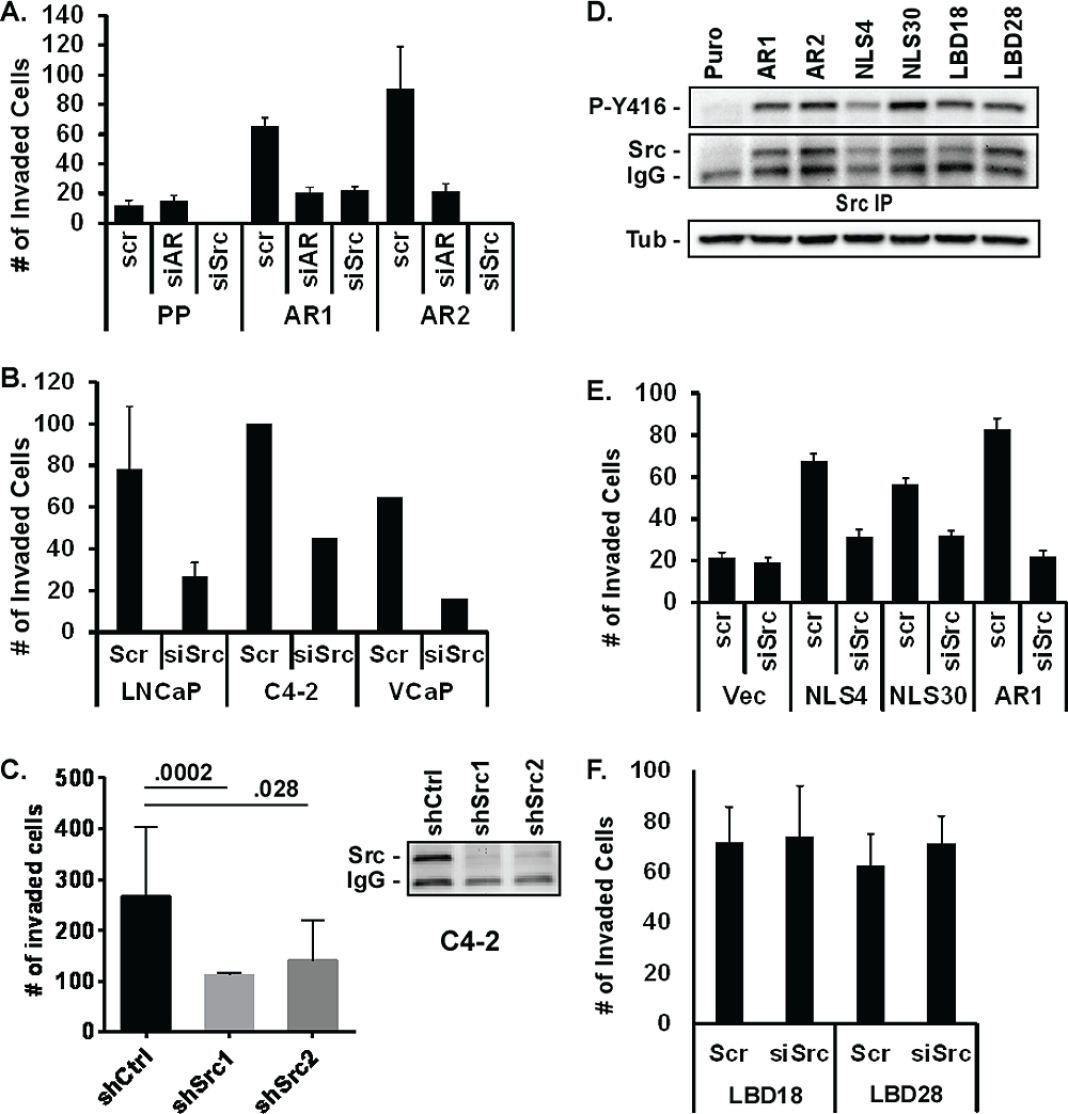
AR and Src are required for invasion **(A)** Matrigel invasion was measured in PC3-Puro (PP) or PC3-AR clones (AR1/AR2) treated with scrambled siRNA (scr), AR-, or Src-specific siRNA (siAR, siSrc). **(B, C)** Matrigel invasion was measured in R1881-stimulated LNCaP, VCaP, or C4–2 cells following treatment with (B) scrambled siRNA (scr) or Src-specific siRNA (siSrc) or upon expression of (C) control shRNA (shCtrl) or two different shSrc constructs. Src levels before and after shSrc expression were measured by immunoblotting of Src immunoprecipitates. **(D)** Src activation and expression was measured in PC3 cell lines expressing wild type (AR1/AR2), nuclear localization deficient (NLS), or ligand binding deficient (LBD) AR mutants by immunoblotting of Src immunoprecipitates with anti-Y416 phospho-specific antibody or total anti-Src antibody respectively. **(E, F)** Matrigel invasion was measured in PC3 (Vec), PC3-AR1, AR-NLS, or AR-LBD clones treated with scrambled siRNA (scr) or Src-specific siRNA (siSrc).

To determine if AR-dependent stimulation of Src is responsible for the increase in invasiveness, cells were transfected with scrambled or specific siRNAs directed towards AR or Src. Inhibition of AR or Src expression attenuated PC3-AR invasion 3- to 4-fold; similar to levels seen in PC3-Puro control cells (Figure 3A). Inhibiting Src expression in LNCaP, C4–2, or VCaP cells with the siRNAs or with individual independent shRNAs similarly decreased R1881-stimulated Matrigel invasion 2.5- to 3-fold (Figure 3B, 3C). To assess whether AR stimulates Src activity and invasion via a non-nuclear mechanism, Src activation was measured in PC3 cells expressing AR with a mutated nuclear localization sequence (ARΔNLS) or AR with a mutated ligand binding domain (ARΔLBD) (Figure 3D). As described previously, the ARΔNLS mutant is cytoplasmic while the ARΔLBD mutant is predominantly nuclear, and both expressed at the same levels as wild type AR in the PC3 cell lines [29, 31, 32]. Src activity and expression were elevated in all AR-expressing mutant cell lines, similar to that seen in PC3-AR cells. Cells expressing the ARΔNLS mutant also displayed increased Matrigel invasiveness compared to vector control cells, which was suppressed 2.5- to 2.8-fold when Src was inhibited with siRNA (Figure 3E). However, despite elevated levels of Src expression and activity in the ARΔLBD mutants, their invasiveness was unaffected by Src inhibition (Figure 3F), indicating that AR ligand binding activity may be crucial for Src-dependent invasion. Thus, the ability of AR to promote Src-dependent invasion occurs via a non-nuclear, but ligand-dependent mechanism.

### AR stimulates Matriptase cleavage and its extracellular shedding via a non-nuclear mechanism

**Figure 4:**
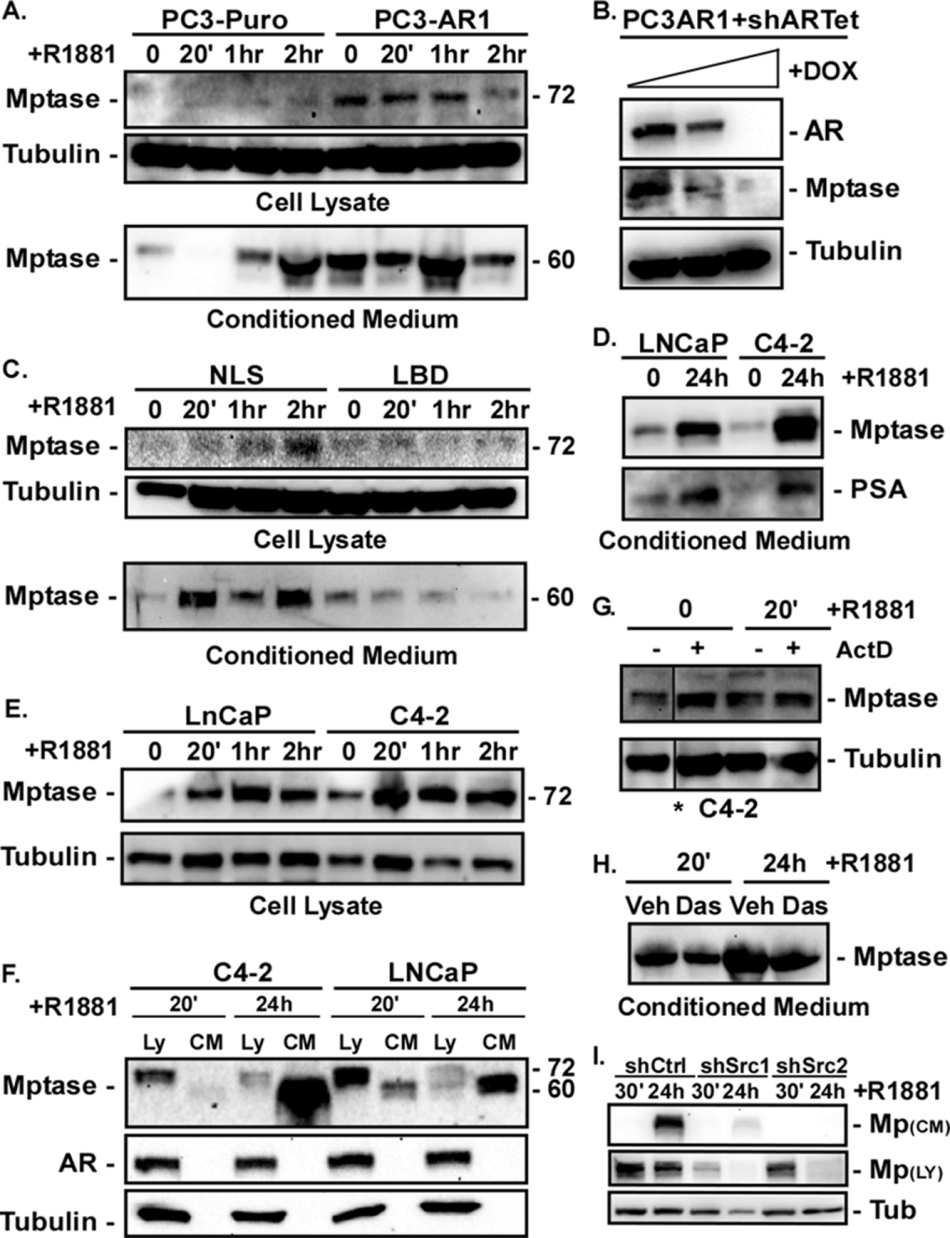
AR stimulates Matriptase activation and extracellular shedding in a non-nuclear fashion **(A)** PC3-Puro or PC3-AR clones were stimulated with 10 nM R1881 for 0, 20, 60, 120 minutes (0, 20′, 1 hr, 2 hr). Levels of active Matriptase in cell lysates and secreted into the conditioned medium were measured by immunoblotting. **(B)** PC3-AR cells stabling expressing a Tet-inducible AR shRNA (shARTet) were stimulated with 100–400 μg/ml doxycycline for 24 hours and the levels of AR and active Matriptase in the cell lysate measured by immunoblotting. **(C)** PC3 cells expressing ARΔNLS or ARΔLBD were stimulated with 10 nM R1881 for 0, 20, 60, 120 minutes (0, 20′, 1 hr, 2 hr). Levels of active Matriptase in cell lysates and secreted into the conditioned medium were measured by immunoblotting. **(D, E)** LNCaP or C4–2 cells were stimulated with 10 nM R1881 for (D) 24 hours or (E) 0, 20, 60, 120 minutes (0, 20′, 1 hr, 2 hr). (D) Active Matriptase secreted into the conditioned medium or (E) in cell lysates was measured by immunoblotting. **(F)** Active Matriptase in the lysates (Ly) or conditioned medium (CM) from LNCaP or C4–2 cells stimulated with 10 nM R1881 for 20 minutes (20′) or 24 hours (24 h) was measured by immunoblotting. **(G)** C4–2 cells were stimulated with ethanol (0) or 10 nM R1881 for 20 minutes (20′) after 2 hours of pretreatment with vehicle (−) or 10 μg/ml Actinomycin D (+). Active Matriptase in the cell lysate was measured by immunoblotting. *All samples were run on the same gel, but image was cut to rearrange lanes. **(H)** C4–2 cells treated with vehicle (Veh) or 10 nM dasatinib for 24 hours were then stimulated with 10 nM R1881 for 20 minutes (20′) or 24 hours (24 h). Active Matriptase in the conditioned medium was measured by immunoblotting. **(I)** C4–2 cells expressing control shRNA (shCtrl) or two different Src shRNAs (shSrc) were stimulated with 10 nM R1881 for 30 minutes (30′) or 24 hours (24 h) and the levels of Matriptase in conditioned medium and cell lysates measured by immunoblotting.

Matrigel invasion requires proteolytic degradation of laminin substrates; therefore, we measured expression of the active form of the laminin protease Matriptase [33–35]. Matriptase is activated by autocatalytic cleavage of the 95kDa zymogen into a transmembrane 72kDa fragment tethered by a disulfide bond to the active enzymatic domain [35]. The active transmembrane-associated protein is then shed as a ~60kd fragment tethered to the active enzyme. Thus, both the cleaved cell-associated and shed enzymes are active. PC3-Puro and PC3-AR cells were stimulated with R1881 for 20, 60 or 120 minutes and the amounts of cleaved cell-associated (cell lysates) and shed (conditioned medium) Matriptase were measured by immunoblotting with an antibody that recognizes the 72kDa transmembrane and 60kd shed fragments. PC3-AR cells displayed elevated levels of cleaved cell-associated Matriptase compared to PC3-Puro cells (Figure 4A). Suppression of AR in PC3-AR cells using a tetracycline-inducible shRNA decreased cleaved Matriptase expression (Figure 4B). Interestingly, despite non-detectable AR expression in PC3-Puro cells, a low level of cleaved Matriptase was detectable in the medium and more was released after 2 hours of androgen stimulation. In contrast, cleaved Matriptase was constitutively shed into the medium of PC3-AR cells. PC3 cells are reported to express low, but unstable levels of AR [36, 37]. Low levels of steroid receptors are often not sufficient to stimulate transcription, but are still capable of mediating nongenomic signaling. That androgen rapidly increases shedding of cleaved Matriptase in PC3-Puro cells suggest a possible non-nuclear mechanism for Matriptase activation when AR expression is low. To test this, we measured cleaved cell-associated and shed Matriptase in PC3 cells expressing the NLS or LBD AR mutant following R1881 stimulation. PC3-ARΔNLS, but not PC3-ARΔLBD, cells induced the accumulation of cleaved cell-associated and shed Matriptase within 20 minutes following androgen stimulation (Figure 4C). Thus, the AR-dependent increase in cleaved Matriptase requires ligand binding, but not nuclear activity.

In complementary experiments with LNCaP and C4–2 cells, Matriptase shedding was observed 24 hours after androgen stimulation (Figure 4D). Cell-associated cleaved Matriptase was elevated within twenty minutes of ligand stimulation (Figure 4E) and remained elevated for a few hours, but by 24 hours its levels decreased while shed Matriptase levels increased (Figure 4F). Thus, androgen initially stimulates Matriptase cleavage within the cell, and that cleaved Matriptase is subsequently shed into the medium. The increase in cleaved Matriptase within 20 minutes of androgen stimulation was resistant to mRNA synthesis inhibition by Actinomycin D (Figure 4G) indicating androgen stimulates this rapid Matriptase cleavage independent of transcription, further supporting a rapid non-nuclear action of AR on Matriptase. Suppressing Src activity with dasatinib or two different Src shRNAs decreased the androgen-dependent induction of Matriptase cleavage in C4–2 cells (Figure 4H, 4I). Thus, the ability of AR to induce Matriptase cleavage and shedding is dependent on Src. Altogether these data indicate AR signaling through Src via a ligand-dependent, but non-nuclear, mechanism is required to stimulate Matriptase cleavage and induce its shedding.

### CDCP1 activity is regulated by AR and Src

**Figure 5:**
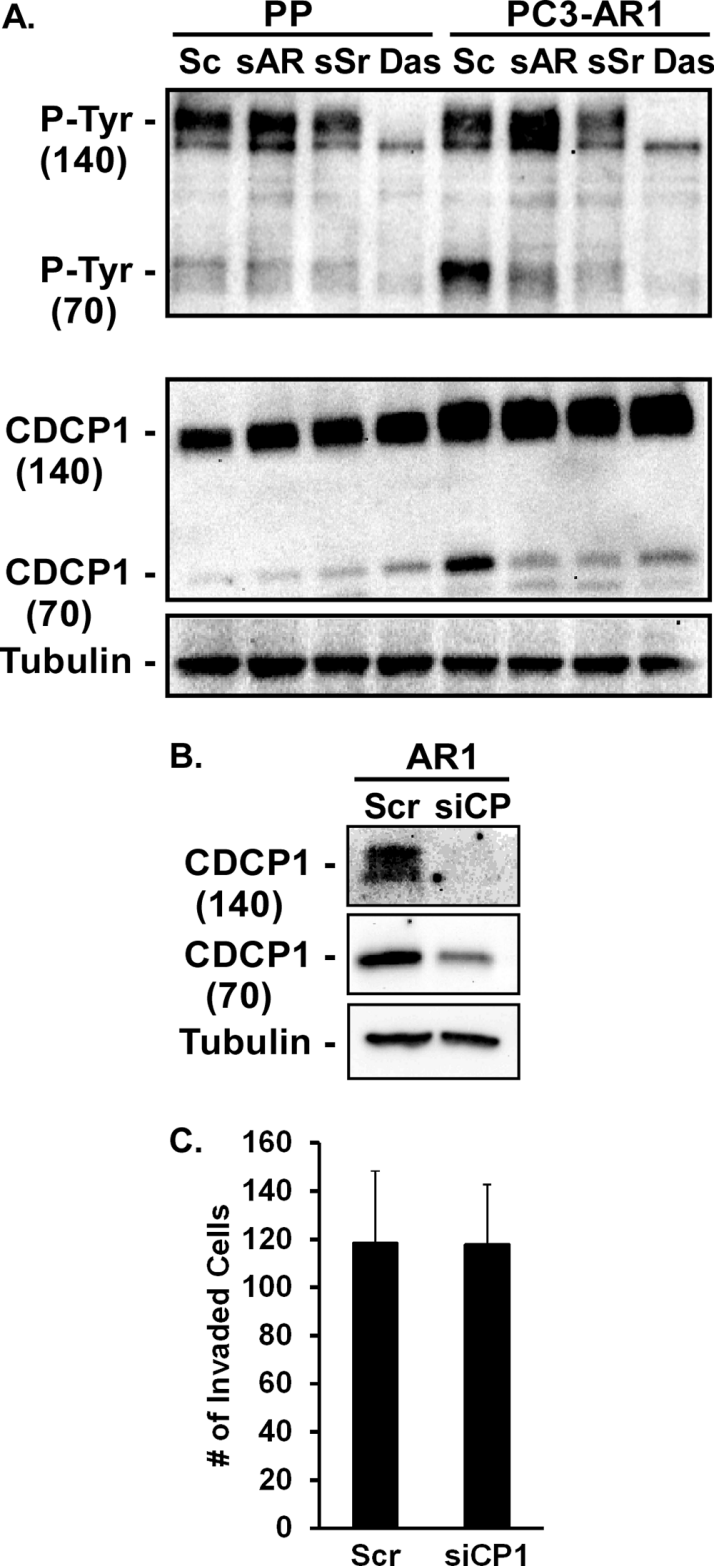
CDCP1 activity is regulated by AR and Src **(A)** PC3-Puro (PP) or PC3-AR cells were treated with scrambled siRNA (Sc), AR- or Src-specific siRNA (sAR, sSr), or 10 nM dasatinib for 24 hours. Tyrosine phosphorylation and expression of full length (140kDa) and cleaved (70kDa) CDCP1 from immunoprecipitates were measured by immunoblotting with anti-phosphotyrosine antibody or CDCP1 antibody respectively. **(B, C)** PC3-AR cells were treated with scrambled siRNA (Scr) or CDCP1-specific siRNA (siCP1). (B) CDCP1 expression was measured by immunoblotting and (C) Matrigel invasion was quantified.

Cub Domain Containing Protein 1 (CDCP1/gp140/Trask) is a transmembrane glycoprotein that facilitates integrin-dependent migration and invasion and whose elevated expression in primary tumors correlates with metastasis in several cancers [38]. CDCP1 is cleaved by several serine proteases, including Matriptase [39]. Full-length CDCP1 (140 kDa) and its cleaved form (72 kDa) are direct Src substrates and phosphorylation by Src is required for promoting cellular de-adhesion from matrix and invasion [40–42]. AR expression in PC3 cells increased the expression, cleavage, and tyrosine phosphorylation of cleaved CDCP1 (Figure 5A). Inhibition of AR or Src expression with siRNA, or treatment with the Src inhibitor dasatinib prevented cleavage and tyrosine phosphorylation of the cleaved fragment (Figure 5A). While dasatinib can target other Src-related tyrosine kinases as well as some receptor kinases, it abrogated generation of the CDCP1 cleavage product (72kDa) and its phosphorylation to a similar extent as the Src siRNA (Figure 5A). Conversely, treatment of C4–2 cells with R1881 rapidly increased CDCP1 cleavage and tyrosine phosphorylation (not shown). However, inhibition of CDCP1 expression (Figure 5B) had no effect on invasion (Figure 5C). Although Src activation by AR is able to induce cleavage and phosphorylation of CDCP1, this target is not critical for AR-dependent invasion of Matrigel.

### Matriptase is required for AR-dependent invasion

**Figure 6:**
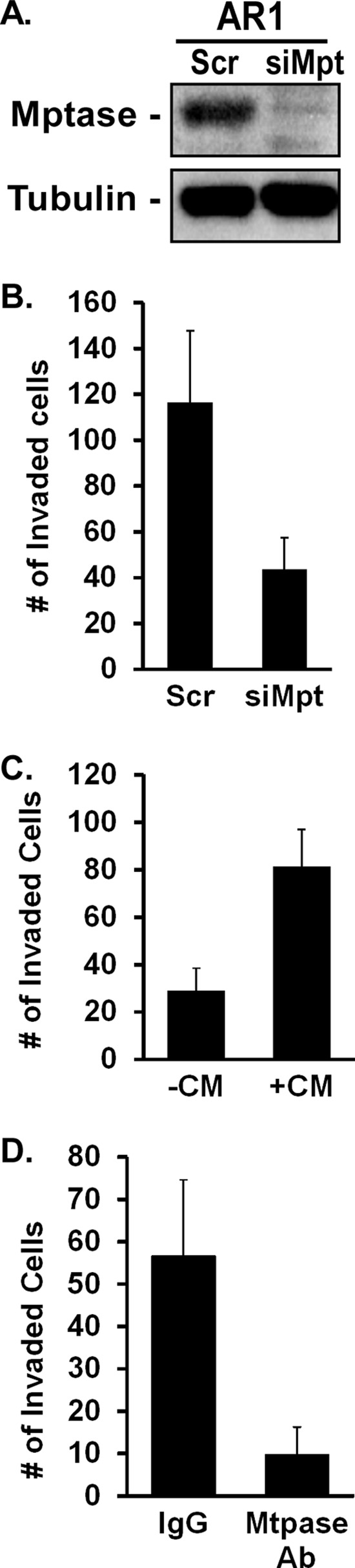
Matriptase, but not CDCP1, promotes AR-dependent invasion **(A, B)** PC3-AR cells were treated with scrambled siRNA (Scr) or Matriptase-specific siRNA (siMpt). (A) Matriptase expression was measured by immunoblotting and (B) Matrigel invasion was quantified. **(C, D)** Conditioned medium (+CM) containing Matriptase from unstimulated PC3-AR cells or BSA (−CM) (C) was added to unstimulated C4–2 cells or (D) CM was first depleted of Matriptase with specific antibody (Mtpase Ab) or IgG and the supernatant added to unstimulated C4–2 cells. The ability of untreated or treated C4–2 cells to invade Matrigel was quantified.

To assess whether Matriptase is required for increased invasion induced by AR signaling, its expression was suppressed by siRNA in PC3-AR cells and the effect on Matrigel invasion evaluated. Inhibition of Matriptase expression (Figure 6A) decreased invasion 3-fold (Figure 6B). Conditioned medium from PC3-AR cells containing cleaved Matriptase was sufficient to increase the invasiveness of androgen-deprived C4–2 cells 3-fold relative to untreated cells (Figure 6C). Conversely, depletion of shed Matriptase from the PC3-AR cell conditioned medium reduced C4–2 cell invasion 5-fold (Figure 6D). Thus, shed Matriptase is required and sufficient to promote Matrigel invasion of prostate tumor cells.

## DISCUSSION

In addition to its role in regulating gene transcription in the nucleus, AR like other steroid receptors rapidly activates signal transduction pathways in the cytoplasm. Pathways reported to be activated by nongenomic AR signaling include MAPK, PI-3K, EGFR, and Src [16, 17, 43, 44]. Functionally, these pathways were linked to AR-dependent increases in cell proliferation. In this study, we demonstrate for the first time that AR stimulates integrin-dependent prostate cancer invasion by stimulating Src activity through a ligand-dependent but non-nuclear mechanism. Src in turn stimulates rapid cell-associated Matriptase cleavage and its extracellular shedding, which is required for laminin matrix-dependent invasion.

We employed several different prostate cancer cell lines to demonstrate the universality of the nongenomic AR/Src invasion pathway. The first model made use of PC3 cells engineered to express AR (PC3-AR). We previously demonstrated that PC3-AR cells display constitutive AR transcriptional activity as measured by nuclear AR localization and elevated PSA and TMPRSS2 expression in the absence of androgen [29]. Cells were always maintained at low passage and grown only in charcoal stripped serum for cultivation to prevent over activation of AR, which often inhibits proliferation as seen in other PC3-AR models. The level of AR expression in these clones was not excessive; comparable to that seen in LNCaP cells as demonstrated previously [29]. LNCaP, C4–2, and VCaP cells that express endogenous AR require androgen to be transcriptionally activated. The advantage of the PC3-AR model is that we were able to express a mutant of AR that is defective in nuclear localization, and thus lacks transcriptional activity, or is defective in ligand binding [29, 31, 32]. Thus, we were able to demonstrate that Src and Matriptase activation and shedding occurs via a nongenomic mechanism, i.e. independent of AR nuclear activity. Additional support for the nongenomic mechanism was afforded by the fact that androgen stimulation of PC3 control cells that do not express detectable levels of AR also stimulates cleaved Matriptase shedding; consistent with the observation that low levels of AR that are not sufficient to activate classical transcriptional responses, are sufficient to activate cytoplasmic signal transduction pathways. We used the androgen responsive lines to demonstrate the AR/Src invasion pathway is also active in androgen-responsive cells. The nongenomic aspects of AR, Src, and Matriptase activation in these cells were evidenced by a rapid response occurring independently of new mRNA synthesis. The lack of dependence of this invasion pathway on the AR-dependent integrin α6 pathway, which is transcriptionally activated by AR [29], further indirectly supports a nongenomic mechanism.

Depending on the model being examined, the ability of steroid receptors to activate nongenomic signaling may or may not be ligand dependent. Our data demonstrating that Casodex and RU486, ligand antagonists, block invasion and Src activation support the conclusion that the AR/Src invasion pathway is ligand dependent. Therefore, we expected the cells expressing the AR ligand binding mutant (LBD) to be neither invasive nor to activate Src. Contrary to expectations, Src was constitutively activated and the cells were highly invasive. However, the PC3AR-LBD cells did not behave like any of the other cell lines in that their invasive activity was not dependent on Src, even though Src activity was elevated; nor did they induce Matriptase activation. Our finding that addition of cleaved and thus activated Matriptase, generated by AR/Src signaling, exogenously to non-stimulated cells was sufficient to increase invasion, indicates that the primary target of AR/Src signaling in invasion is Matriptase. Thus in the PC3AR-LBD cells, the failure to activate Matriptase in spite of active Src, suggests it is Matriptase activation that is sensitive to ligand binding. It further suggests that depending on whether Src is activated through a ligand-dependent or -independent mechanism may determine which downstream targets are affected and the biological outcome.

Our data strongly support a nongenomic mechanism for Matriptase activation. It occurs in the absence of nuclear function and in the absence of new mRNA synthesis. A previous study demonstrated that androgen induces Matriptase activation and shedding in LNCaP cells 6–24 hours after stimulation [45]. In that study, Matriptase mRNA was elevated 24 hours after androgen stimulation and was dependent on new mRNA and protein synthesis at that time point. Combining those findings with ours, we envision a model whereby nongenomic AR signaling to Src, either through direct phosphorylation of Matriptase or indirectly through an associated molecule, triggers an initial activation of the autocatalytic cleavage of Matriptase between 20 minutes and 2 hours. The cell-associated Matriptase diminishes at the same time it appears in abundance in the conditioned medium, over the course of 24 hours (Figure 4F). The cleavage and release of Matriptase depletes cellular stores, triggering an AR-dependent, but indirect, transcriptional increase in Matriptase mRNA as reported previously [45].

To determine additional aspects of the mechanism by which AR/Src signaling to Matriptase increases invasion, we interrogated CDCP1, a known integrin regulator and Src and Matriptase substrate [38–42]. While CDCP1 cleavage and phosphorylation was dependent on AR and Src, it was not required for AR-dependent invasion. Clinically, this is important because CDCP1 expression is often elevated in primary cancers and prognostic of poor outcome and survival [46]; however, recent evidence indicates its expression actually decreases within metastatic prostate cancer lesions (B. Knudsen, personal communication). While, CDCP1 may be necessary *in vivo* for initial metastatic dissemination [47], the dependence on CDCP1 in late stage disease may decrease. It is also possible that CDCP1 dependency is specific to a subset of integrin/matrix interactions.

While enzalutamide can extend patient survival for 6 months, it is far from curative [48]. One mechanism proposed for enzalutamide resistance is increased Src activation [18]. Whether this is mediated through cytoplasmic-localized AR, AR variants that arise during enzalutamide resistance [49], or by some other mechanism remains to be determined. Our data further indicate that when Src is activated in resistant disease it could activate Matriptase. In laminin-enriched tumor microenvironments, such as that found in the bone and lymph nodes [50], Matriptase activation may enhance metastatic spread and could serve as an ideal therapeutic target in resistant disease.

## MATERIALS AND METHODS

### Cell culture

The prostate tumor cell lines PC3, LNCaP, and VCaP, were purchased from American Type Culture Collection. C4–2 cells were obtained from Dr. Robert Sikes, University of Delaware [51]. PC3 cells were grown in F-12K media supplemented with 10% charcoal-stripped and charcoal-stripped fetal bovine serum (CSS), 2 mM glutamine, 50 U penicillin, and 50 μg/ml streptomycin. LNCaP, VCaP, and C4–2 cells were grown in RPMI 1640 media (Invitrogen) supplemented with 10% fetal bovine serum, 2 mM glutamine, 50 U penicillin, 50 μg/ml streptomycin, 0.225% glucose, 10 mM HEPES, and 1 mM sodium pyruvate. For experiments, LNCaP, VCaP, and C4–2 cells were seeded on laminin (Millipore) and grown in phenol-red free media and 0.1% CSS for 24 hours before and throughout the experiment. All cells were grown at 37°C in 5% CO_2_.

### DNA constructs and cell lines

Stable clonal isolates of PC3 cells expressing empty vectors, PC3-Puro and pLKO.1, or wild type or mutant AR, PC3-AR, PC3-ΔNLS, and PC3-ΔLBD (N705S), were generated by infecting cells with retroviruses or lentiviruses as described previously [29]. PC3-AR Tet-ON shRNA clones were generated by using pLKO-Tet-ON vector (Novartis) that contained a single AR shRNA, 5′-CCGGCCTGCTAATCAAGTCACACATCTCGAGA TGTGTGACTTGATTAGCAGGTTTTT-3′, purchased from Open Biosystems and cloned upstream of the H1/TO promoter as described [52, 53]. Src shRNAs, shSrc1: 5′-GACAGACCTGTCCTTCAAGAA-3′ and shSrc2: 5′-GCGGCTCCAGATTGTCAACAA-3′ in TRC pLKO vector were purchased from Sigma. The AR Tet-ON and Src shRNA plasmids were sequence validated and packaged into lentiviruses using 293FT cells (Invitrogen). PC3-AR cells were infected with Tet-ON-ARshRNA or Src shRNA lentiviruses and individual clones were selected using 1–3 μg/ml puromycin.

### siRNA transfections

A pool of four small interfering RNAs (siRNA) against androgen receptor (siGENOME SMARTpool), integrin α6 (ON-TARGETplus SMARTpool); integrin α3 (ON-TATRGETplus SMARTpool); Src (ON-TARGETplus SMARTpool); CDCP1 (ON-TATRGETplus SMARTpool), or a non-targeting sequence were purchased from Dharmacon. Matriptase-specific siRNA was obtained from Santa Cruz Biotechnology, Inc. Serum-deprived sub-confluent cells were transfected with siRNA using siLentFect lipid reagent (Bio-Rad) and Opti-MEM (Invitrogen) medium following manufacturer's directions. The medium was changed 16 hours after siRNA transfection. All pools were titrated to determine the lowest optimal concentration for inhibition of protein expression by immunoblotting 72 hours after transfection.

### Drug treatments

Mifrepristone (RU486) was purchased from Tocris Bioscience (Ellisville, MO). Dasatinib was a gift from Dr. Matt Steensma (Van Andel Research Institute). Bicalutamide (Casodex) was purchased from Enzo Life Science (Farmingdale, NY). Each drug was diluted into ethanol and used at a final concentration of 10 nM. Metribolone (R1881) was purchased from PerkinElmer (Boston, MA). R1881 was diluted into ethanol and then into phenol red free media and used at a final concentration of 10 nM in all experiments. Actinomycin D was purchased from Calbiochem and reconstituted in DMSO at a concentration of 10 μg/mL. For R1881 treatments, cells were starved 24 hours in 0.1% charcoal-stripped serum (CSS) prior to stimulation. Inhibitors were added during the starvation period and maintained during androgen stimulation.

### Immunoblotting

Total whole cell lysates were prepared for immunoblotting as previously described [30, 54]. Briefly, cells were lysed on ice with MAPK lysis buffer (50 mM Tris pH 7.5, 0.5 mM EDTA, 50 mM NaF, 100 mM NaCl, 50 mM β-glycerophosphate, 5 mM sodium pyrophosphate, 1% Triton-X100, 1 mM Na_3_VO_4_, 1 mM PMSF, 5 μg/ml leupeptin, 5 μg/ml pepstatin, 10 μg/ml aprotinin, 1 mM benzamide) or RIPA (10 mM Tris pH 7.2, 158 mM NaCl, 1 mM EDTA, 0.1% SDS, 1% NaDOC, 1% Triton-X100, 1 mM Na_3_VO_4_, 1 mM PMSF, 100U/ml aprotinin, 10 μg/ml pepstatin and 10 μg/ml leupeptin). For immunoblotting, 40–65 μg of total cell lysates in 2X SDS sample buffer were boiled for 5 minutes and run on SDS polyacrylamide gels following standard SDS-PAGE protocols, then transferred to PVDF membranes. Membranes were blocked in 5% BSA in TBST for two hours at room temperature, and then were probed with primary antibody for two hours at room temperature. Signal was detected, after incubation with horseradish peroxide-conjugated secondary antibodies (Bio-Rad) in 5% BSA in TBST for 1 hour at room temperature, by chemiluminescence with a CCD camera in a Bio-Rad Chemi-Doc Imaging System using Quantity One software v4.5.2 (Bio-Rad).

Primary antibodies were as follows: anti-AR monoclonal antibody (Santa Cruz Biotechnology); anti-Bcl-xL polyclonal antibody (Cell Signaling Technologies); anti-Matriptase polyclonal antibody (Bethyl Laboratories); anti-phospho-[Y^416^]-Src polyclonal antibody (Invitrogen); anti-Src monoclonal antibody (Src 327) [55]; anti-CDCP1 polyclonal antibody (Cell Signaling Technologies); and anti-Tubulin monoclonal antibody (Sigma-Aldrich).

### Immunoprecipitation

For detection of phosphorylated CDCP1 and phospho-[Y^416^]-Src, cell lysates were prepared as described above. Protein samples (500 μg), in a volume of 500 μL of 1X MAPK buffer, were incubated with 1 μg of mouse monoclonal anti-Src antibody [55] or anti-CDCP1 (Cell Signaling Technologies) overnight at 4°C. Protein-antibody complex was precipitated with protein G or protein A beads (Thermo Scientific) respectively followed by immunoblotting with rabbit anti-phospho-[Y^416^]-Src antibody (Invitrogen) or mouse 4G10 anti-phospho-tyrosine antibody (Millipore) as described above.

### Immunofluorescence

Cells were fixed with 4% paraformaldehyde in PBS at 4°C for twenty minutes and permeabilized for ten minutes with TBS (10 mM Tris, pH 8.0, 150 mM NaCl) + 0.5% TritonX-100 at room temperature. Cells were then blocked with 2% BSA in TBS + 0.1% Triton X-100 for twenty minutes at room temperature before incubation with vinculin antibody (Sigma) and Alexafluor 546-Phalloidin (Molecular Probes, Invitrogen) for one hour. Cells were incubated with goat anti-mouse Alexa Flour-488 secondary antibody for one hour at room temperature. DNA was visualized by staining with Hoechst 33258 for 10 minutes at room temperature. Cells were washed four times with TBS + 0.1% TritonX-100 over ten minutes between all steps. Epifluorescent images were acquired on a Nikon Eclipse TE300 fluorescence microscope using OpenLab v5.5.0 image analysis software (Improvision).

### Migration assays

Cell migration was measured using the modified Boyden chamber assay. Cells (5 × 10^4^) were suspended in the upper well of the 8.0 μm pore size polyethylene terephthalate membrane culture inserts for (BD Biosciences) in which the bottom membrane was pre-coated with 1 μM laminin. The lower and upper chambers were filled with 400 μL phenol-red and serum-free DMEM. After 6 hours of incubation, the culture inserts were removed and washed with 1X PBS. Cells that had migrated to the lower membrane surface were stained with Crystal Violet (Millipore) and counted under a microscope in five random fields per insert in triplicate.

### Matrigel invasion assays

Serum-starved sub-confluent cells (6.25 × 10^4^) were suspended in the upper well of the 8.0 μm pore size Matrigel membrane culture inserts (BD Biosciences) in which the bottom membrane was pre-coated with 1 μM laminin. The upper and lower chambers were filled with 400 μL phenol-red and serum-free DMEM. After 72 hour incubation, the culture inserts were removed and washed once with 1X PBS. Cells that invaded through Matrigel to the lower membrane surface were stained with Crystal Violet (Millipore) and counted under a microscope in five random fields per insert in triplicate.

### Isolation of conditioned medium

To examine Matriptase shedding into the medium, cells were washed 2 times with PBS and placed in serum-free medium for 24 hours prior to R1881 stimulation. 10–15 mL of medium was collected on ice from the plates and loaded into Amicon Ultra-15 centrifugal filter units (Millipore). The Amicon filter tubes were centrifuged at 4000 × g for 30 minutes at 4°C. Concentrated medium was collected and subjected to SDS PAGE and transferred to PVDF membrane for immunoblotting. Protein loading was normalized to total cell number.
